# Ecological Compensation Strategy for SDG-Based Basin-Type National Parks: A Case Study of the Baoxing Giant Panda National Park

**DOI:** 10.3390/ijerph17113908

**Published:** 2020-05-31

**Authors:** Chenyang Xue, Chaofeng Shao, Junli Gao

**Affiliations:** 1 College of Environmental Science and Engineering, Nankai University, Tianjin 300071, China; 2120170641@mail.nankai.edu.cn (C.X.); 17854232337@163.com (J.G.); 2 Guangzhou Research Institute of Environmental Protection, Guangzhou 510620, China

**Keywords:** ecological compensation, national parks, sustainable development goals, basin, payment for ecosystem services

## Abstract

An ecological compensation mechanism is the basic condition for the sustainable development of national parks and the key institutional measure to implement goals 1, 3, 6, 10, 12, 13, 15, 16, and 17 of the sustainable development goals. In this study, the current ecological compensation mechanism was summarized and analyzed from the aspects of promotion mode, realization routine, and implementation effect, on the basis of the sustainable development needs of national parks and the public welfare character in construction and management. In addition, the practical demands of ecological compensation for basin-type national parks were presented in the setting of the main body and mode of multiparticipation, and the key points of compensation. The “1 + 1 + *N*” basin-type national park ecological compensation system was designed on the basis of the framework of horizontal protection and vertical development. Taking the Baoxing Giant Panda National Park as an example, typical compensation scenarios were designed from five common compensation approaches; namely, fund, project, technology, material, and policy compensations. The compensation modes were selected and the effect was predicted in combination with local actual situation. Finally, the optimal combination scheme of ecological compensations for national parks was determined on the basis of the return on investment index.

## 1. Introduction

The United Nations put forward 17 sustainable development goals (SDGs) in September 2015, which aim to mobilize global efforts to end poverty, foster peace, safeguard the rights and dignity of all people, and protect the planet [1]. The SDGs call for deep transformations in every country, that will require complementary actions by governments, civil society, scientific research institutions and enterprises. Sachs proposed a plan for implementing the SDGs, and identified six integrated transformations to achieve all the SDGs [2]. In transformation 4, which is broadly focused on sustainable land use and agricultural systems, achieving the sustainable management and efficient use of natural resources by 2030 is the content of SDGs [3,4]. Planet and partnership are two keywords of SDGs, which means that it is not only necessary to establish a self-enforcing win-win partnership between human and nature, but also between stakeholders [5]. However, as suggested in TWI2050 Report [6] and Global Sustainable Development Report 2019 [7], vested interests, regulatory capture and other factors are challenges and obstacles to implementing transformations and SDGs. According to the current major assessment reports on the progress of sustainable development [8,9,10,11], the SDGs related to ecological environment in various countries are generally poor. The scores of goals 1, 3, 6, 10, 12, 13, 15, 16 and 17 are generally low, the research progress is slow, and the evaluation results of these indicators are far from the requirements of sustainable development, which is the key constraint for most countries, especially developing countries, to achieve the SDGs. The reason is that the ecological environment protection mainly depends on the government’s passive promotion, but lacks the mechanism of the whole society’s active response. Compensation strategies have already been proposed as a solution to overcome these problems, so the compensation problem must be considered firstly, to build the better frameworks for partnerships among winners, losers, and orchestrator. In addition to implementing new governance policies, vested interests should also be incentivized to align their actions with compensation [12].

Compensation is used broadly in economics to mitigate the negative effects of disruptions in certain areas, such as immigration [13], trade liberalization [14], technical change [15], and climate policy [16]. Environmental conservation is the founding objective of ecological compensation programs. Ecological compensation programs induce environmental service providers to adopt sustainable land use practices to protect ecosystems and their associated services, such as water filtration, carbon sequestration, and protection of wildlife habitats [17], by providing appropriate incentives. However, the objective should also include poverty alleviation and economic development, because environmental service providers in developing countries are often poor and rely on environmental services supplied by the ecosystem to maintain their livelihood [18,19]. In developing countries, environmental conservation programs elicit little appeal unless packaged as part of poverty relief measures [20]. Thus, the ecological compensation mechanism, as an economic instrument for balancing the interest relationship between the protector and the beneficiary of the ecological environment, has become the core content of ecological civilization construction and the important link in achieving goals 1 (No poverty), 3.9 (Reduce the number of deaths and illnesses from environmental pollution), 6 (Ensure availability and sustainable management of water and sanitation for all), 10 (Reduce inequality within and among countries), 12.2 (Sustainable management and efficient use of natural resources), 13 (Climate action), 15 (Halt biodiversity loss), 16.6 (Develop effective, accountable and transparent institutions) and 17 (Strengthen the means of implementation and revitalize the global partnership for sustainable development). Current studies on ecological compensation in the world focus on the concept and theory of ecological compensation [21,22], compensation principle [23], compensation space selection [24], compensation subjects and objects [25], compensation standards [26,27,28], compensation methods [29], compensation performance evaluation [30], and other aspects that involve river basin, forest, grassland, farmland, wetland, ocean, mineral resources, and other fields.

Among the objects of ecological compensation, national park is the most special because it is a strictly controlled area for biodiversity conservation in various countries. However, the development opportunities of national park residents are considerably restricted. Local people living in close proximity to the parks bear many costs and risks, despite the government’s objective of using tourism development for rural poverty elimination. For instance, national parks and other nature reserves are predominantly located in rural areas. This placement limits land for agriculture, irrigation, and housing and restricts the development activities of rural residents. In addition, crop and livestock depredation by wild animals, inflation due to tourism, disruptions to social networks and local culture, and external investment pressures create costs and risks for the local economy. New compensation methods are required because of the irreplaceable, scarce, and special management of national parks.

Currently, China has 10 declared national parks, which cover 222,900 km^2^ and are under the management of the central government. Overall, the establishment of ecological compensation systems for national parks in China remains in the initial development stage, and problems, such as the one-sided compensation subject and the solidified compensation model, still exist. To protect the ecosystem integrity of national parks, current compensation methods are all under fund compensation to implement “strict protection and prohibition of development”. All revenues collected from national parks are transferred to the central government’s treasury, and the surrounding communities are typically not allowed to gain substantial benefits, including the overextraction of park resources and the illegal use of the natural assets of parks. The share that goes to local residents through the park value chain is questionably small and does not conform to the concept of sustainable development. Simultaneously, the current research is limited to the top-level design of systems and mechanisms, and actual scenario prediction is not available. Thus, selecting the optimal combination is impossible. Accordingly, the Giant Panda National Park in the Baoxing River Basin was selected as the research object in this study because most of the national parks with rich biodiversity are located in the river source regions or areas with abundant water sources. Development scenarios are comprehensively designed and predicted under different compensation approaches, by combining the management mechanism of a national park and basin characteristics. The compensation approach with equal emphasis on protection and development is explored, and the “1 + 1 + *N*” compensation system is constructed to maximize the benefits of ecological compensation in providing scientific support for the improvement of ecological compensation mechanisms of national parks in China and other countries.

## 2. Research Progress

### 2.1. Experience and Insight into the Ecological Compensation of Typical River Basins and Nature Reserves in China and Other Countries

Watershed ecological compensation is a popular topic in the field of ecological compensation. This compensation is also the research direction with the most practice and has been highly valued by countries and international organizations worldwide. For example, the ecological compensations of the Elbe River Basin in Germany through the government’s financial transfer, the Rhine Meuse Watershed in France through one-to-one market trading between mineral water companies and farmers, the Delaware River in the United States through the collection of resource tax, the Darling River in Australia on the basis of salt credit [31,32], and other typical ecological compensation models, constitute the mature ecological compensation mechanism of a river basin, in which the government and the market coexist. In addition, the ecological compensation of nature reserves is primarily achieved using two approaches: national financial expenditure and tourism industry development.

Existing ecological compensation mechanisms and models of national parks lack attention to public welfare. Thus, summarizing and analyzing social realization models in the ecological compensation mechanisms of international river basins and nature reserves in accordance with the needs of the social public welfare attributes of national parks are urgently required. The ecological compensation cases of typical river basins and nature reserves in China and other countries are listed in Table 1.

As shown in Table 1, from the perspective of compensation subjects, the social public is an important participant and component of ecological compensation subjects under the appropriate guidance of the government and the market. From the perspective of compensation approaches, the economic compensation is combined with the production factors compensation. Service, project, and technical compensations are receiving increasing attention in promoting the self-regulation of a compensated area to achieve sustainable development. The economic compensation refers to the realization of ecological and economic prosperity in the compensated area by means of external compensation, such as cash payments and stock options [5], that is, the traditional payments for ecosystem services [42]. The production factors compensation refers to the formation of the self-development mechanism and the finding of a new economic growth point, based on the protection of ecological environment through the innovation of industrial technology and the adjustment of industrial structure [43]. The coupling of their advantages can be used as reference to establish an ecological compensation model of a basin-type national park in China.

### 2.2. Realistic Requirements for Developing Ecological Compensation for Basin-Type National Parks

(1) The participation channels for compensation subjects should be broadened.

The characters of a national park, such as national representativeness and public welfare, cause reliance on the government for ecological compensation [44]. At present, the compensation fund source for national parks only relies on the vertical transfer of government finance and results in the lack of participation of stakeholders, such as downstream beneficiary areas and social compensation subjects.

(2) Compensation methods should be enriched, and development opportunity compensation should be prioritized.

At present, the ecological compensation implemented in national parks remains only at the levels of fund compensation and environmental protection project compensation, and a series of losses, such as the loss of development opportunities of economically backward ecological counties, is not fully considered. This situation does not satisfy the requirements of SDGs. Therefore, the economic compensation should be improved, and the production factors compensation should be enriched and strengthened, to provide more development opportunities for residents.

## 3. Framework of SDG-Based Ecological Compensation System for Basin-Type National Parks

**Figure 1:**
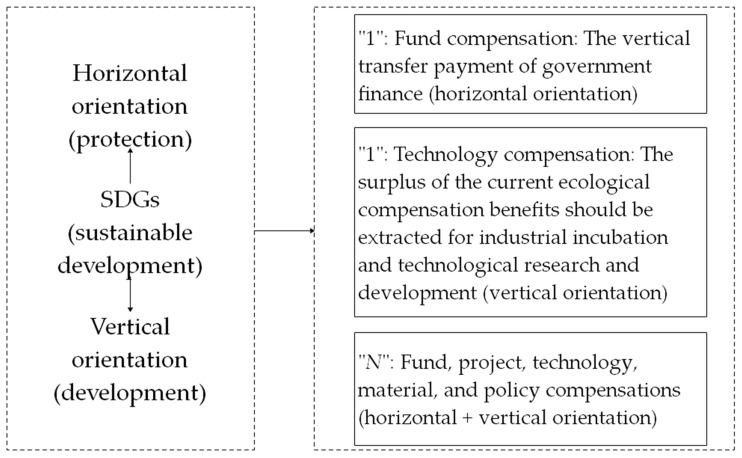
Sustainable development goals (SDG)-based ecological compensation system for basin-type national parks.

**Figure 2:**
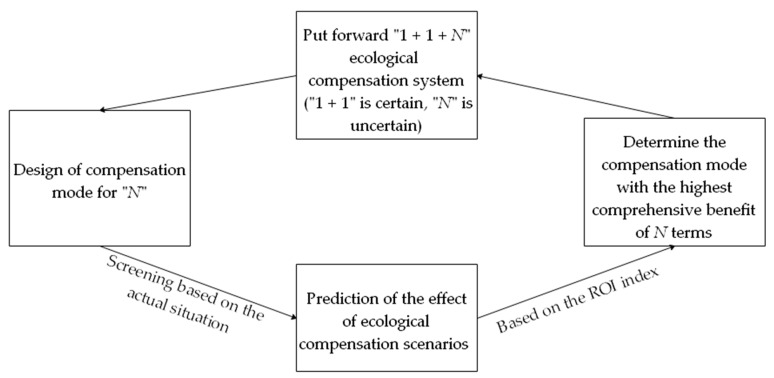
The specific model work flow chart.

A national park is established and managed by the state. It is the wealth of the entire population as a special ecological environment area. On the basis of the stakeholder and the game theories [45,46], compensation subjects include social citizens besides governments, enterprises, and individuals in the upstream and downstream areas. Considering the relevant requirements of Goals 1, 3, 6, 10, 12, 13, 15, 16 and 17 in the SDGs, this study has built an overall framework in the horizontal and the vertical orientations, set protection as the goal in the horizontal orientation, and explored diversified ecological compensation approaches to improve the efficiency of economic compensation. Sustainable development is considered the goal in the vertical orientation. The fulcrum of social and economic development is innovated to improve the green development mechanism, and the “1 + 1 + *N*” SDG-based ecological compensation system for basin-type national parks (Figure 1) is established. In this system, “1 + 1” refers to “the vertical transfer payment of government finance” in the horizontal orientation and “the withdrawal of a part of current benefits for technological research” in the vertical orientation. These processes are necessary comprehensive compensation approaches. “*N*” refers to selective and targeted compensation approaches that should be designed in combination with the actual situation of the research area and can be enriched and expanded from the perspectives of fund, project, technology, material, and policy compensations. In this paper, a new method of green accounting or a new model of green growth for natural resource management are presented on the basis of the design of the compensation model, which is using people’s willingness to pay or market prices to estimate the ecological value of natural resources [47], then different compensation scenarios will be predicted and the benefit will be judged on the basis of the return on investment (ROI) index [48]. The actual expected financial return on the ecological compensation programs is provided to the decision-makers in advance before the values for ecosystem services are obtained, to determine the priority of policy implementation. The specific model work flow chart is shown in Figure 2.

## 4. Design of SDG-Based Ecological Compensation Mode for Basin-Type National Parks

### 4.1. Research Area

The combined zone of the Sichuan Basin and the Qinghai Tibet Plateau, where the Baoxing County is located, is the Hengduan Mountains, one of the top 10 biodiversity centers in the world. The Hengduan Mountains are known as the “sanctuary of the world’s endangered animals and plants” and a “global important ecological region”, as determined by the World Wide Fund for Nature [49]. The Baoxing County is where pandas are first discovered, and more than 90% of its territory has been incorporated into the Giant Panda National Park. This national park is highly important for the protection of the world’s fauna and flora gene pool. However, mining and hydropower developments are the two major pillar industries in the area, and the current management position of the national park has considerably restricted its development opportunities. Balancing the relationship between protection and development with the help of an ecological compensation mechanism is crucial for the construction of the Baoxing Giant Panda National Park.

### 4.2. Design of Compensation Mode

Various compensation approaches under “1 + 1 + *N*” are analyzed and explained, and the design of the selective compensation mode is expanded by considering the actual situation of the Baoxing Giant Panda National Park.

1: Vertical transfer payment of government finance. The main source of funds for national park protection in most countries is government finance. In the current government budget, 22.4 million yuan has been invested for the construction of the protection and utilization facilities in the Baoxing Giant Panda National Park. Considering all the national characteristics and public welfare attributes of the park and to ensure ecological security in the national security system, the vertical transfer payment of government finance will be continued, in the short term and in the future.

1: The surplus of the current ecological compensation benefits should be extracted for industrial incubation and technological research and development. The current development should leave room for future improvement. Thus, technical and intellectual compensations should be implemented. However, the source of funds should be extracted from the region’s current ecological compensation benefits to a certain percentage, to create new economic growth points continuously, optimize industrial layout, and innovate social production factors. The extraction ratio can be set to 12% by using the investment of basic research funds and relevant research in developed countries as reference [50].

*N*: Nine types of selective ecological compensation scenarios, including fund, project, technology, material, and policy compensations, are designed in accordance with the actual situation of local development in the Baoxing County. These scenarios guarantee the protection of the natural ecosystem of the Giant Panda National Park and consider the requirements of sustainable development to provide new production factors of social and economic development.

(1) Conversion of cropland to forest land: fund compensation

Providing subsidy for the conversion of cropland to forest land is a mature method in China’s ecological compensation system [51,52]. In 2019, the Baoxing County government provided 0.59 million yuan of ecological compensation funds to farmers who have completed the conversion of 254.7 ha of cropland to forest land. This compensation method can be applied to national park construction in the future, because most national parks in China have ecological functions.

(2) Ecological restoration for mines: fund compensation

A fund pool for ecological compensation should be established by collecting resource taxes and deposits from the developers of mineral resources, in accordance with the principle of “who destroys, who recovers, who pollutes, who handles, who benefits, who pays.” In addition, the government should act as the representative of resource owners to recover and handle mines with a historical legacy.

(3) Water rights trading: fund compensation

The river chief system of China promotes the implementation of horizontal ecological compensation mechanism in the river basin. The natural resources in the national park are owned by the country. Thus, the river chief system is used to conduct water rights trading between the upstream and the downstream governments. Market-oriented methods are used to achieve a multifaceted promotion of ecological compensation for each river.

(4) Construction of environmental protection infrastructure: project compensation

The Baoxing County can emulate the water quality betting compensation mechanism of the Xin’an River, with the goal of ecological environment protection [53]. Setting outbound water quality as the evaluation standard and relying on the river chief system, the financial horizontal transfer payment is conducted with downstream city governments, or a green project credit is implemented in cooperation with banks, to gain the commitment of the entire basin to the construction of the environmental protection infrastructure and continuously improve water quality.

(5) Technical transformation of mining: technology compensation

The Baoxing County government should provide technical transformation support for mining. Collecting only the environmental protection and restoration deposit from enterprises for the ecological compensation of mining in accordance with the principle of “who destroys, who repairs” is insufficient. This method limits the development of enterprises and is unsustainable for the ecological environment. Therefore, the government should provide technological compensation for enterprises, innovate the new production factors of social and economic development, and realize development under protection.

(6) Creating an eco-label: material compensation

Local agriculture, forestry, medicine, fishing, and animal husbandry resources should be selected to strengthen their utilization and development research. A standardized production technology and market-oriented service system should be established to achieve industrialized operation and management, and the advantages of local resources should be changed into economic advantages. For example, a rare species, such as the *Schizothorax prenanti* in the Baoxing River, can be used to create the China Protected Geographical Indication Products and the Giant Panda National Park Indication Products. This compensation approach is listed as material compensation, because this article analyzes enhancement and releasing, which means that fries are placed in the river.

(7) Selling water and electricity at low prices: material compensation

In addition to implementing new governance policies, vested interests should also be incentivized, to align their actions with compensation. The farmers upstream must sacrifice development opportunity to ensure the safety of drinking water and the quantity of water resources. Therefore, under the regulation of the government, water companies and hydropower stations downstream can implement the dual-track pricing strategy [12], and compensate the farmers at the water source area, by selling water and electricity at low prices to ensure their basic livelihood.

(8) Attempting enclave economy: policy compensation

Following the off-site development model between Pan’an County and Jinhua City [54], the industrial parks that belong to the upstream city should be built in the downstream city on the basis of the government’s promotion. Simultaneously, the upstream city should refuse polluting enterprises to ensure the safety of the environment of the water source area. All the taxes obtained by the enterprises in the new development zone should be used as compensation for ecological services in the upstream city.

(9) Providing policy support for the development of the eco-tourism industry: Policy compensation

The Baoxing County should rely on its rich natural resource advantages and the brand effect of the Giant Panda National Park, cater to the current social psychological characteristics of high-quality life and spiritual pursuit, adjust the local industrial structure, vigorously develop comprehensive rural eco-tourism and forest health tourism, promote the demonstration park of agriculture and animal husbandry, and create a characteristic town. The Baoxing County should flexibly introduce ecological education products, such as farming, mulberry planting, silkworm breeding, fruit and vegetable picking, inland river sightseeing, water conservancy sightseeing, and aquaculture sightseeing; develop an artificial intelligence virtual tour, a beach camp, an outdoor mountain club, and other high-end tourism industries; and establish ecological product businesses that rely on “Internet+” and online live broadcasting. The new vitality of regional economic development will be activated through preferential tax policies and the establishment of startup funds, to fully realize the organic integration of primary, secondary, and tertiary industries and achieve the coordinated and sustainable development of ecology, economy, and culture, to form its own green development mechanism.

### 4.3. Prediction of the Effect of Ecological Compensation

#### 4.3.1. Selection of the Scenario Prediction Model of Ecological Compensation

From the perspective of protection and development, scenarios 1 and 2 protect the ecological environment and increase the ecological assets by improving vegetation coverage. Ecological resources can be transformed into ecological assets through water rights trading (scenario 3), the improvement of production technology (scenario 5), and the enhancement of product value (scenario 6), which have economic benefits. Scenario 4 and scenario 7 achieve the protection of water environment and water resources, through the establishment of environmental protection infrastructure and low-cost water and electricity sales. Scenarios 8 and 9 have social benefits by ensuring people’s livelihoods.

**Figure 3:**
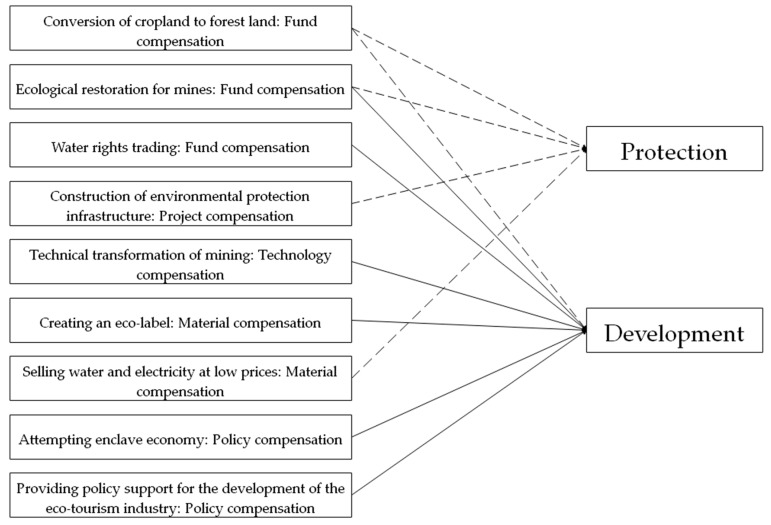
Selection of scenario prediction model of ecological compensation.

The quantifiable compensation mode with economic development benefits is selected for analysis in the scenario prediction of ecological compensation. Six compensation modes, namely, scenarios 2, 3, 5, 6, 8, and 9, are selected for scenario prediction, because of the uncertainty of the area of the cropland to be converted into forest in scenario 1 and the operability of the six scenarios in the quantitative model for predicting the effect of ecological compensation (Figure 3). The operation cycle for the ROI analysis is set to five years. It is worth noting that calculations of ROI for different scenarios in this paper are predictions of future development, not assessments of the measures that have been taken. As shown in Figure 3, the solid line represents the scenario to be simulated in this study, and the dotted line represents the scenario that cannot be quantified at present.

#### 4.3.2. Prediction of the Effect of Ecological Compensation

(1) Scenario 2: Ecological restoration for mines

(a) Investment: According to the standards set by the Chinese government, the ecological restoration fund for mines is 0.153 million yuan/ha. The mining sites assigned to the ecological restoration area in the Baoxing County are 263.8 ha. Thus, approximately 40.3614 million yuan needs be invested.
(b) Return: The value of the forest ecosystem service function in the mining ecological restoration area is predicted from four aspects; namely, environment purification, water conservation, nutrient recycling storage, and water and soil conservation, by using the shadow project method [55], the market value method [56], and referring to the relevant prices in “Specification for assessment of forest ecosystem service in China,” which has been published by the State Forestry Administration [57]. The prediction results are presented in Table 2.

Based on a 5-year operating cycle, the final ecological benefit is 106.6833 million yuan.

(2) Scenario 3: Water rights trading

The runoff of the Baoxing River is 3.346 billion m^3^. At present, the development and utilization of water resources in the Baoxing County only account for 1.23% of the total amount of the county, and the internationally recognized threshold is 40%. Therefore, the potential tradable water resources of the Baoxing River except those for local development account for 1.297 billion m^3^. According to the China Water Exchange Company Limited, the average trading price is 0.6 yuan/m^3^ [58]. Thus, the potential water exchange value of the Baoxing River is 778 million yuan.

(3) Scenario 5: Technical transformation of mining

(a) Investment: The technical transformation support fund provided by the local government for the expansion of mining and other projects within five years, based on the development plan for major project construction in the Baoxing County, is 3.343 billion yuan.
(b) Return: The Baoxing County started to increase the investment in mining technology transformation projects in 2012. The additional benefits of the mining industry with technology transformation in five years can reach approximately 34.295 billion yuan on the basis of a growth rate of 25%, in accordance with the growth rate of the mining industry in the statistical yearbook of the Baoxing County for the last five years and in consideration of the restrictive effect of the construction of the Giant Panda National Park on industrial development.

(4) Scenario 6: Creating eco-labels

(a) Investment: Approximately 50,000 rare fish fries should be placed annually in the Baoxing River, with a cost of approximately 0.15 million yuan in accordance with the actual local experience data [59]. Approximately 0.75 million yuan will be invested in an operation cycle.
(b) Return: The market value of eco-labels in an operation cycle is approximately 6 million yuan, on the basis of the growth rate of local fish and the price of local specialty products.

(5) Scenario 8: Attempting enclave economy

Different stakeholders can gain different benefits. For enterprises, relocating heavy industry enterprises to downstream industrial parks for development and changing the original extensive operation to intensive management is conducive in promoting the optimization of the industrial structure and improving production efficiency and economic benefits. For local residents, reducing pollution sources is highly important downstream and to the improvement of the local social environment and the construction of a harmonious and symbiotic ecosystem. For the Baoxing County government, the enclave economy can satisfy the assessment requirements of environmental performance and cannot restrict the development of local pillar industries. Thus, the enclave economy can effectively balance economic development and environmental protection, achieve a win–win situation in “ecological environment gross domestic product (GDP)” and “economic GDP,” and satisfy the requirements for sustainable development.

(6) Scenario 9: Providing policy support for the development of the eco-tourism industry

In recent years, the proportion of the tertiary industry in the Baoxing County has continuously increased annually. The Baoxing County can find a new foothold through the adjustment of industrial structure for sustainable economic development, which solves livelihood problems (such as employment); promotes the targeted poverty alleviation; improves public satisfaction and the residents’ happiness index; fully unearths and spreads local history and ecological culture; and finally creates a “city card” of the Baoxing County.

The summary of each compensation scenario is provided in Table 3. In this study, the ROI is used to predict the benefits of ecological compensation in the Baoxing County. ROI is expressed as:

(1) $$\mathrm{ROI}\;=\frac{K}{IN}\;,$$

where K represents the total investment, and IN represents the total benefit. A small ROI indicates that the project has a good economy.

## 5. Results and Discussion

Considering the geographical location of the National Park, this paper conducts research on SDG-based ecological compensation model for basin-type national parks, puts forward the concept of ecological compensation with equal emphasis on protection and development, and designs the “1 + 1 + *N*” compensation mechanism. Two kinds of necessary comprehensive compensation approaches and nine kinds of selective and targeted compensation approaches are designed by coupling the national park management mechanism, watershed characteristics and the actual situation of the Baoxing Giant Panda National Park. By considering the actual local situation and the availability of technology comprehensively, the scenario design and the benefit prediction are further selected for the selective compensation approaches, and the ecological compensation scheme that can be carried out as a priority is determined on the basis of the ROI index. The scenarios in this study are virtual for ecological compensation, not policies that have been implemented. For the Baoxing Giant Panda National Park, water rights trading can use natural resources directly to obtain economic benefits; technical transformation of mining can reduce raw material consumption and increase product output compared with before technical transformation; eco-labels can add value to products. According to the calculation results of the ROI index, the economic and ecological benefits of them are the most substantial for the Baoxing Giant Panda National Park, meet the requirements of sustainable development, and belong to the first best solution. Therefore, the “1 + 1 + 3” ecological compensation mechanism of the Baoxing Giant Panda National Park can be built, together with the vertical transfer payment of government finance and the withdrawal of a part of the current benefits for technological research. National and local actual conditions should be considered comprehensively for the selection of “*N*” in the ecological compensation approach of other basin-type national parks.

Accordingly, developing countries, such as China, need to redefine their objectives for national park management and should be reoriented toward sustainability (inclusive of socioeconomic sustainability), rather than focusing only on the conservation of natural assets. Ecological compensation as an environmental conservation and poverty alleviation instrument is becoming increasingly popular in achieving the given goals. Therefore, this paper establishes the “1 + 1 + *N*” ecological compensation system for basin-type national parks. However, scrutinizing its potential and predicting the compensation effect are necessary to determine the priority of policy implementation. In this study, the feasible alternatives are compared on the basis of the ROI index, and the actual local situation and the availability of technology are considered comprehensively to identify and screen ecological compensation methods, so as to continuously improve the sustainable development evaluation indicators scores and green development ability of national parks and other important ecological functional areas. For an ecological compensation scenario, its ROI analysis needs to be considered from many aspects. Thus, the application of simple variables to a scenario prediction is limited, and a more comprehensive prediction needs to be carried out in the future. In addition, this research focuses on basin-type national parks, and further studies on the level of sustainability of other types of national parks and natural reserves are needed.

## 6. Conclusions

In accordance with the concept of sustainable development, ecological compensation is used to balance the relationship between the protection and development of national parks and other natural reserves, and scenario prediction is carried out, in which green accounting model is used to assess the value of natural resources and the ROI index is used to assess the comprehensive benefits, to maximize the benefits, and to achieve the purpose of scientific decision-making for the government.

## Figures and Tables

**Table 1:** Ecological compensation cases of typical river basins and nature reserves in China and other countries.

State/Region	Protected Object	Content	Compensation Mode	Compensation Approaches
Several countries	National parks	More than 100 international environmental protection organizations exist worldwide and provide financial support to different national parks in the world [33]. Examples include the World Wide Fund for Nature (Canada), the Canadian Nature Foundation, and the Sierra Club [34].	Social voluntary donation	Fund compensation
China	Chishui River Basin	Four wineries have committed to invest 0.5 billion yuan as ecological compensation fund for the prevention of water pollution in the Chishui River Basin for 10 consecutive years since 2014 [35].	Social voluntary donation	Fund compensation
Ecuador	Cayambe Coca Watershed	The water resource protection fund, which is managed by a professional organization that is independent of the government, is established to protect the land and ecosystem upstream.	Government compensation, market transaction	Fund, project, and technical compensations
United States	Delaware River	The New York City government subsidizes environmental protectors in upstream areas to encourage dairy and forest farm owners to adopt environment-friendly production methods.	Public payments led by the government	Fund compensation (mostly from the collection of taxes), the issuance of New York City bonds, and trust funds.
England	/	The government issues lottery tickets with low-carbon and environmental protection attributes to invest in carbon reduction and emission reduction projects, such as a wind power plant in Turkey, a hydropower station in India, and green energy communities in Brazil and Guatemala [36].	Public payments led by the government	Fund compensation (mostly from lottery ticket buyers)
China	Three-river source region	The theme sports lottery is issued, and 35% of the revenues are used as public welfare fund to realize the combination of public welfare sports lottery and ecological environment protection [37].	Public payments led by the government	Fund compensation (mostly from lottery ticket buyers)
New Zealand	New Zealand National Park	A franchise operation model is established, and tourism projects, such as hunting, horse riding, kayaking adventure, and helicopter sightseeing, are launched on the premise of obtaining a license [38].	Public payments led by the government and the market	Fund compensation (mostly from tourists)
China	Qiandao Lake and seven water sources	The Nongfu Spring Company provides a penny in each bottle of water through brand promotion and a differentiated marketing method to compensate the people in the water source area [39].	Public payments led by the market	Fund compensation (mostly from the added value of ecological products)
United States	Yosemite National Park	More than 10,000 volunteers provide construction and maintenance, education, vegetation restoration, wildlife protection, camp maintenance, and other services every year [40].	Volunteer services led by the government and nonprofit organizations	Service compensation
China	Taroko National Park, Yangmingshan National Park	Interpreter, conservation, and trail volunteers are recruited to serve the national parks and reduce expenditures [41].	Volunteer services led by the government	Service compensation

**Table 2:** Prediction of ecological service function in the mining ecological restoration area.

Ecosystem Service Function		Evaluation Method	Functional Capacity per Unit	Total Functional Capacity	Value per Unit	Total Value (Thousand Yuan/A)
Environment purification	Carbon sequestration	Carbon tax method	15.75 t/hm^2^·a	4154.85 t/a	1200 yuan/t	4985.8
	Oxygen release	Industrial treatment cost method	11.51 t/hm^2^·a	3036.34 t/a	1000 yuan/t	3036.3
	SO_2_ absorption		0.15 t/hm^2^·a	40.10 t/a	1200 yuan/t	48.1
	Dust retention		21.15 t/hm^2^·a	5579.37 t/a	150 yuan/t	836.9
Water conservation		Shadow project method	993.70 mm/a	262,138.06 m^3^/a	5.48 yuan/m^3^	1436.5
Nutrient recycling storage		Market value method	0.10 t/hm^2^·a	27.24 t/a	2500 yuan/t	69.4
Water and soil conservation	Reduce soil erosion		/	1592.78 hm^2^/a	282.17 yuan/hm^2^	449.4
	Reduce sediment deposition		/	1,911,333.57 m^3^/a	5.48 yuan/m^3^	10,474.1
Summary		/	/	/	/	21,336.7

**Table 3:** Comprehensive analysis of the ecological compensation scenarios.

No.	Scenario	Compensation Approach	Compensation Subjects	Compensated Subjects	Benefit Type	Investment	Return	ROI
2	Ecological restoration of mines	Fund compensation	Local government, enterprises	Environment	Economic benefits	40.3614 million yuan	106.6833 million yuan	2.64
3	Water rights trading		Downstream government	Upstream government		/	778 million yuan	/
5	Technical transformation of mining	Technology compensation	Local government	Enterprises		3.343 billion yuan	34.295 billion yuan	10.26
6	Creating eco-labels	Material compensation	Local government	Residents		0.75 million yuan	6 million yuan	8
8	Attempting enclave economy	Policy compensation	Downstream and upstream governments	Enterprises	Social benefits	Establishment of industrial parks downstream for off-site development	Achieve a win–win result among residents, enterprises, governments, and the natural ecosystem	/
9	Providing policy support for the development of the eco-tourism industry		Local government	Residents		Industrial restructuring		/
